# MicroRNAs as a potential missing link between environment and type 1 diabetes development

**DOI:** 10.3389/fimmu.2026.1943501

**Published:** 2026-10-19

**Authors:** Sebastian Ciężki, Milena Jamiołkowska-Sztabkowska, Magdalena Walicka, Artur Bossowski, Barbara Głowińska-Olszewska

**Affiliations:** 1 Clinical Department of Internal Medicine, Endocrinology and Diabetology, National Medical Institute of the Ministry of the Interior and Administration, Warsaw, Poland; 2 Department of Pediatrics, Endocrinology, and Diabetology with Cardiology Division, Medical University of Białystok, Białystok, Poland; 3 Mossakowski Medical Research Institute, Polish Academy of Sciences, Warsaw, Poland

**Keywords:** autoimmunity, environmental triggers, epigenetic regulation, microRNA, type 1 diabetes

## Abstract

The pathogenesis of type 1 diabetes (T1D) is multifactorial and remains incompletely understood. Although genetic susceptibility plays a major role, epidemiological evidence indicates an important contribution of non-genetic factors. Viral infections, dietary factors, alterations in gut microbiota, pollutants and early-life exposures have all been associated with T1D risk, but the molecular mechanisms linking these exposures to disease development remain unclear. MicroRNA dysregulation has been reported in T1D and is associated with immune regulation, pancreatic β-cell function, insulin secretion and cell survival. At the same time, environmental and early-life exposures can alter microRNA expression. These two lines of evidence raise the hypothesis that environmentally responsive microRNAs may represent molecular points of convergence between environmental influences and pathways relevant to T1D. In this narrative review, we examine evidence linking environmental and early-life exposures to microRNA alterations and evaluate the potential relevance of these changes to T1D-related immune and β-cell pathways. Across human studies and T1D-relevant experimental models, environmental and early-life exposures have been associated with alterations in microRNA expression, with several microRNAs recurring across distinct environmental contexts. Among these, miR-155, miR-146a and miR-375 emerge as candidate convergent regulatory nodes linking environmental responses with immune and β-cell pathways relevant to T1D. However, direct evidence that environmentally induced alterations in these microRNAs mediate T1D development in humans is still lacking. These findings may also have clinical relevance. Circulating microRNA signatures could complement existing biomarkers in T1D, but consistent analytical methods and replication in independent cohorts are still needed before routine use. Therapeutic targeting of microRNAs remains experimental. Overall, the reviewed evidence points to environmentally responsive microRNAs as candidate molecular links between environmental exposures and immune and β-cell pathways relevant to T1D, although a causal role in disease development has not been established.

## 1 Introduction

Despite decades of intensive research and medical advances, type 1 diabetes (T1D) remains a disease that not only significantly reduces quality of life, but also carries the risk of serious, often life-threatening complications, which may shorten life expectancy (1, 2). Epidemiological predictions suggest a significant increase in the incidence of T1D in the near future (1), which also applies to the youngest patients (3, 4). T1D arises from an interaction of genetic, immune, and environmental factors, but its precise pathogenic mechanisms have yet to be fully clarified (5).

Although the contribution of gene polymorphisms to the disease is fairly well established, environmental determinants and their underlying mechanisms continue to be inadequately understood despite intensive scientific research (6).

It is known that genetic susceptibility is a key contributor to T1D development, with up to 50% of the risk attributable to inherited factors (7). In the course of extensive research, it was demonstrated that polymorphisms in the HLA (human leukocyte antigen) region on chromosome 6p21 account for approximately half of the hereditary risk of type 1 diabetes (8, 9). Polymorphisms within HLA class II (HLA-DRB1, HLA-DQA1, HLA-DQB1) loci are considered the primary genetic risk factor, whereas a comparatively reduced effect is attributed to variation in HLA class I (HLA-A, HLA-B, HLA-C) genes (8, 9). The majority of genetic risk is associated with specific high-risk HLA class II haplotypes, such as DR3-DQ2 and DR4-DQ8, which are found in approximately 90% of patients with T1D (10).

As previously mentioned, HLA haplotypes are critically involved in the predisposition to T1D development, but they do not account for the entire genetic risk. This finding suggests the involvement of non-HLA-dependent factors such as single nucleotide polymorphisms (SNPs) identified in genes encoding preproinsulin, the insulin precursor protein (INS), protein tyrosine phosphatase - one of T-cell regulators (PTPN22), alpha subunit of the IL-2 receptor (IL2RA) and cytotoxic T-lymphocyte associated protein 4 – inhibitory immune checkpoint (CTLA4) (11–15). In addition, genes implicated in thymic T cell development (e.g. *PTPRK* and *THEMIS*) are involved as well (16). What is more, numerous polymorphisms in more than 60 non-HLA loci have been identified by genome-wide association studies as potentially influencing susceptibility to T1D (17).

Interestingly, over the past four decades, a decline in the prevalence of classic high-risk HLA genotypes among children diagnosed with T1D has been observed (18). In view of the rising global incidence of T1D, this phenomenon suggests that genetic susceptibility is not a sufficient determinant of T1D development. Rising incidence, incomplete twin concordance, geographic, migratory, and age differences in disease prevalence indicate that other non-genetic factors, including environmental ones, may play a significant role in T1D pathogenesis (19).

Studies indicate that multiple environmental factors such as viral infections, exposure to pesticides, dietary and lifestyle habits, vitamin D deficiency, alterations in gut microbiota and even climatic factors may contribute to the increased risk of developing T1D (20), potentially through epigenetic and other molecular mechanisms, although the pathways linking these exposures to disease development remain incompletely understood (18).

In recent years, emerging evidence has suggested that microRNAs, short RNA molecules without coding potential, may serve as potential molecular links between environmental stimuli and immune and β-cell pathways involved in T1D, mainly by regulating gene expression (21).

This review aims to highlight the impact of environmental factors on the development of T1D, focusing on how changes in microRNA regulation influenced by external stimuli may affect immune and metabolic pathways relevant to disease development.

## 2 Literature search strategy

The literature search for this narrative review was initiated in August 2025 and updated through September 2026. PubMed, Embase and Scopus were searched as the principal bibliographic databases, with Google Scholar used as a supplementary source to identify additional relevant publications. The search focused on environmental and early-life exposures that had previously been investigated in relation to T1D risk, islet autoimmunity or disease development, and on their potential associations with microRNA dysregulation.

Search terms combined concepts related to type 1 diabetes, microRNAs and T1D-relevant environmental exposures. These included combinations of (“type 1 diabetes” OR “T1D” OR “islet autoimmunity”) AND (“microRNA” OR “miRNA” OR “miR”) together with exposure-related terms, including “viral infection”, “enterovirus”, “Coxsackievirus”, “diet”, “vitamin D”, “polyphenols”, “breastfeeding”, “maternal”, “pregnancy”, “birth weight”, “gut microbiota”, “microbiome”, “dysbiosis”, “antibiotic”, “air pollution” and “PM2.5”. Additional targeted searches were performed when necessary to identify studies addressing specific microRNAs and their links to immune regulation, inflammatory signaling, intestinal barrier function, β-cell survival, insulin production and secretion, and other pathways relevant to T1D.

English-language, peer-reviewed publications were considered, whereas preprints were excluded. Studies were included when they examined: (1) environmental or early-life exposures previously associated with T1D risk or islet autoimmunity and their relationship with microRNA expression; (2) dysregulation of the same or related microRNAs in T1D or established islet autoimmunity; or (3) functional effects of these microRNAs on pathways relevant to T1D. Human studies, animal studies and *in vitro* experiments were considered because direct studies assessing the complete environmental exposure–microRNA–T1D pathway within the same population or experimental setting remain limited. Relevant reviews were used to provide background and to identify additional primary studies, whereas primary studies were prioritized for specific exposure–microRNA associations and mechanistic claims.

Reference lists of relevant publications were also manually screened for additional studies. Environmental exposures without a previously described association with T1D or islet autoimmunity were outside the predefined scope of the review.

Because this was a narrative rather than a systematic review, study selection was guided by relevance to the predefined conceptual scope rather than by a formal systematic-review protocol.

## 3 MicroRNAs as regulators of gene expression

First identified in 1993, microRNAs are small, non-coding RNAs of about 22 nucleotides that play a pivotal role in regulating gene expression (22). The identification of microRNAs was such a groundbreaking discovery that it was awarded the Nobel Prize in 2024 (23). Their biogenesis ultimately leads to the formation of the microRNA-induced silencing complex (miRISC) (24), which by associating with its target mRNAs promotes translational repression and enhances mRNA decay (25). Each microRNA has the capacity to modulate a multitude of mRNAs, thereby exerting extensive regulatory effects on a broad range of cellular functions (26), such as proliferation, differentiation and apoptosis (27).

While the majority of studies emphasize the inhibitory role of microRNAs on gene expression, a subset has described their ability to upregulate expression of target genes (24, 28, 29).

Altered microRNA expression has also been implicated in autoimmune diseases, including T1D (30) and thyroid diseases in pediatric populations (31). By facilitating B cell differentiation and influencing T cell development, homeostasis, and activation, specific microRNAs may regulate immune function (30).

## 4 MicroRNAs and type 1 diabetes

Dysregulated microRNA expression has been implicated in T1D pathogenesis through effects on pancreatic β-cell function, insulin production and immune regulation (32).

In T1D, β-cell destruction is driven by autoreactive CD8^+^ and CD4^+^ T cells recognizing β-cell–derived antigens (33). In this process, CD4^+^ T cells provide essential help to effector CD8^+^ T cells, enhance antibody production by B cells, and promote the activation of pancreatic macrophages (33).

MicroRNAs are important regulators of immune cell development, proliferation and activation (32).

miR-142-3p has been implicated in islet autoimmunity through impaired regulatory T-cell differentiation and stability (34). miR-142-3p targets the epigenetic regulator TET2 and alters Foxp3-associated DNA methylation, thereby impairing Treg stability (34). Guay et al. showed that lymphocyte-derived exosomes transferred miR-142-3p, miR-142-5p and miR-155 to pancreatic β-cells, promoting pro-inflammatory chemokine expression and apoptosis (35).

Zurawek et al. found miR-487a-3p to be upregulated in children with newly diagnosed T1D and identified the immune-regulatory genes *CTLA4* and *FOXO3* as its targets (36).

Furthermore, plasma miR-409-3p levels were reduced in individuals with newly diagnosed T1D and inversely correlated with HbA1c, while reduced miR-409-3p expression was also observed in islet-infiltrating immune cells from non-obese diabetic (NOD) mice (37).

Other microRNAs may influence the persistence and function of autoreactive T cells. In T1D-derived autoreactive CD8^+^ T-cell clones, miR-23b, miR-98 and miR-590-5p were among the upregulated microRNAs and targeted components of the FAS/FASL and TRAIL/TRAIL-R extrinsic apoptotic pathways. This impaired pro-apoptotic signaling and favored the survival and expansion of diabetogenic cytotoxic T cells (38).

MicroRNA dysregulation has also been identified in CD4^+^ T-cell subsets from individuals at increased risk of T1D. In particular, increased miR-26a expression in activated Tregs was associated with reduced expression of EZH2, an epigenetic regulator involved in maintaining the Treg programme, including FOXP3 expression. Experimental inhibition of EZH2 reduced Treg-associated gene expression, supporting a potential link between altered microRNA expression and impaired immune regulation before clinical T1D (39). The same study identified additional alterations involving miR-101, miR-15a and miR-31 in specific CD4^+^ T-cell and Treg subsets, indicating broader microRNA dysregulation of T-cell function during the preclinical stage of T1D (39).

Dysregulation of microRNAs has also been implicated in impaired insulin production and secretion.

miR-30d promotes insulin production by suppressing MAP4K4, a kinase that negatively affects MafA, an important transcriptional regulator of the insulin gene (40). In contrast, miR-9 can reduce insulin secretion by decreasing Onecut2 and consequently increasing Granuphilin, a protein that restrains insulin-granule release from β-cells (41).

miR-25 has also been linked to insulin regulation. In rat β-cell models, miR-25 directly targeted the 3′UTR of Ins1 mRNA, with miR-25 overexpression reducing insulin expression and its inhibition enhancing insulin synthesis and secretion under glucose stimulation (42). Circulating miR-25 was also increased in children with newly diagnosed T1D and was associated with residual β-cell function and glycemic control (43).

Accumulating evidence also suggests that microRNAs can directly affect pancreatic β-cell survival and function. In an inflammatory β-cell model, IL-1β increased miR-18 expression, while miR-18 overexpression promoted apoptosis and impaired insulin production and secretion. These effects were accompanied by direct suppression of NAV1 (neuron navigator 1), a miR-18 target in β-cells, and reduced PI3K/AKT signaling (44).

Certain microRNAs may additionally serve as markers of β-cell loss. miR-204 is highly enriched in human β-cells and is released during β-cell death. Serum miR-204 was increased in individuals with T1D and in autoantibody-positive individuals at risk of T1D and was inversely associated with residual β-cell function, supporting its potential use as a circulating marker of β-cell loss (45).

Despite growing insight into the role of microRNAs in T1D pathogenesis, the mechanisms initiating their dysregulation remain incompletely understood. Among the candidate upstream triggers, environmental factors have attracted considerable attention because they can alter microRNA expression across different tissues and experimental settings. Therefore, we aimed to synthesize the current evidence linking environmental exposures to microRNA dysregulation and explore how these alterations may affect immune and β-cell pathways relevant to T1D development.

## 5 Bridging environment and type 1 diabetes: the role of microRNAs

Understanding how environmental factors influence cellular mechanisms potentially relevant to type 1 diabetes is important for elucidating its etiology. MicroRNAs, as post-transcriptional regulators responsive to a wide range of environmental stimuli (46), may represent potential molecular links between environmental exposures and immune, metabolic and β-cell pathways involved in T1D.

The following section examines evidence linking environmental exposures associated with T1D to changes in microRNA expression and considers how these alterations may contribute to the modulation of pathways involved in immune regulation and β-cell function. Although the available evidence varies considerably in strength and often derives from different tissues, species and experimental settings, recurrent associations between environmental exposures and microRNAs involved in T1D-related processes support their potential role in linking environmental influences with disease-relevant biology.

Environmental factors associated with microRNA dysregulation and their potential relevance to T1D-related pathways are summarized in **Table 1**. **Figure 1** highlights microRNAs reported in association with more than one environmental exposure, suggesting potential convergence of different environmental influences on recurrent microRNA-regulated pathways relevant to T1D, although this does not establish a shared causal mechanism.

**Table 1:** Overview of environmental and early-life factors associated with microRNA dysregulation and their potential relevance to type 1 diabetes, classified by the T1D relevance of evidence for the factor–microRNA association.

Environmental factor	Dysregulated microRNA(s)	Model and tissue	Sample size	Evidence category	Direction	Proposed target/pathway	Proposed context-dependent consequence/inference boundary
Higher maternal pre-pregnancy BMI with lower gestational weight gain	miR-146a (51), arm not specified	Human placenta; female-offspring analysis	n = 211 mother–newborn pairs; female-offspring analysis n = 112	C	Lower expression in girls at low gestational weight gain; association was not significant at higher (~≥14 kg) gestational weight gain	TRAF6, IRAK1/NF-κB signaling (52)	Potential weakening of negative feedback on inflammatory signaling. *The ENVIRONAGE study assessed placental miR-146a, not T1D or β-cell outcomes; the inflammatory mechanism derives from independent mechanistic literature.*
Gestational hypertension and preeclampsia	miR-210-3p (55)	Maternal plasma-derived exosomes	Study n = 98; groups relevant to this row: controls n = 34, gestational hypertension n = 14, moderate preeclampsia n = 15, severe preeclampsia n = 19; chronic hypertension n = 16.	C	Higher expression; >2-fold upregulation in moderate and severe preeclampsia	FOXP3, directly regulated by miR-210 in independent Treg study (58)	Potential impairment of Treg-mediated immune tolerance. *Transfer of this specific maternal circulating miRNA to the fetal circulation was not assessed, and the link to offspring T1D remains hypothetical.*
Hashimoto’s thyroiditis (maternal relevance inferred)	miR-21-5p (60)	Human serum	n=24 with HT; controls n=17	C	Upregulation	BCL2 (62); *Tgfb2 and Smad2* (63)	β-cell apoptosis through BCL2 suppression and loss of β-cell identity/function through Tgfb2/Smad2 targeting were shown in separate β-cell/islet studies *The miRNA data were obtained in affected adults, not during pregnancy or in offspring.*
Inflammatory bowel disease (maternal relevance inferred)	miR-21-5p (64)	Human intestinal mucosa, multiple cohorts	IBD n = 5–41; controls n = 5–35 across included cohorts	C	Upregulation	BCL2 (62); *Tgfb2 and Smad2* (63)	Potential β-cell apoptosis and loss of β-cell identity/function are based on mechanistic studies conducted outside the IBD cohorts. *The IBD miRNA studies did not examine pregnancy, offspring or T1D risk.*
	miR-155-5p (64)	Human intestinal mucosa, multiple cohorts	IBD n = 8–24; controls n = 8–23 across included cohorts	C	Upregulation; most consistently reported in ulcerative colitis	NF-κB; CCL2, CCL7, CXCL10 (36); SOCS1–JAK/STAT axis (66)	Potential amplification of inflammatory signaling and β-cell injury. *These downstream mechanisms derive from T1D β-cell and diabetic kidney disease models; maternal/offspring relevance is inferential.*
Higher birth weight (macrosomia)	miR-375 (68), arm not specified	Human neonatal dried blood spots	n=17–20 per group	C	Upregulation	PDK1-AKT (70), Psen1–Notch1/NICD1 signaling (71), Pc, Pdk4 and Mdh1 (72)	Potential effects on β-cell survival, differentiation and glucose-stimulated insulin secretion. *These mechanisms were established in pancreatic stem-cell, INS-1 and islet studies, not in the neonatal birth-weight cohort.*
Coxsackievirus B5 infection	miR-155-5p (76)	Human pancreatic islets, *in vitro*	n= 2 donors	B	Upregulation	GLIS3 – predicted target (76)	Potential relevance to GLIS3-associated β-cell survival. *GLIS3 mRNA was not significantly altered in infected islets, and direct miR-155-5p–GLIS3 regulation was not validated in this model.*
	miR-34a-5p (76)	Human pancreatic islets, *in vitro*	n= 2 donors	B	Upregulation	VAMP2, BCL2 (77) (mechanistic study reported miR-34a without specifying the mature arm)	Reduced VAMP2/GSIS and BCL2-associated β-cell apoptosis were demonstrated after miR-34a manipulation in MIN6 cells. *These functional effects were not directly tested in CVB5-infected human islets.*
Persistent Coxsackievirus B4 infection	miR-204-5p (79)	Human pancreatic ductal cell line (PANC-1), *in vitro*	n = 4 infected + 4 controls	D	Upregulation	MafA — directly validated target of miR-204 in β-cell/islet models (80)	Potential reduction of insulin transcription/production. *The miR-204–MafA mechanism was established in β-cell/islet models, including human islets, and was not tested in persistently infected PANC-1 cells.*
	miR-146a-5p (79)	Human pancreatic ductal cell line (PANC-1), *in vitro*	n = 4 infected + 4 controls	D	Upregulation	IRAK1, TRAF6; type I interferon signaling (81) (EV71 model)	Potential attenuation of antiviral type I interferon responses. *Direct IRAK1/TRAF6 regulation and restoration of IFN-β after miR-146a inhibition were demonstrated in EV71 models, not in the CVB4-infected PANC-1 system.* *The upregulation in miR-146a-5p contrasts with the downregulation observed after PM2.5 exposure, indicating context-dependent regulation across different exposures and tissues; see Section 7 for discussion.*
	let-7f-1-3p, let-7b-3p and let-7d-3p (79)	Human pancreatic ductal cell line (PANC-1), *in vitro*	n = 4 infected + 4 controls	D	Downregulation	Predicted T1D-risk genes: AFF3, GLIS3 and NAA25 (let-7b-3p/let-7f-1-3p); RASGRP1 (let-7f-1-3p) (79)	Potential perturbation of regulation of predicted T1D-risk genes. *Functional metabolic consequences were not established for these specific 3p species in the infected cells.*
	miR-10a-5p (79)	Human pancreatic ductal cell line (PANC-1), *in vitro*	n = 4 infected + 4 controls	D	Downregulation	Treg-associated regulatory phenotype (83) (murine miR-10a study)	Possible relevance to autoimmune regulation. *The Treg association derives from separate mouse data and was not examined in CVB4-infected PANC-1 cells.*
Coxsackievirus B4 infection	miR-AMC1 (84)	Human pancreatic β-cell line EndoC-βH1, *in vitro*	n = 3 biological replicates	B	Upregulation	Predicted targets enriched in insulin/IGF–MAPK signaling; LGI3 (predicted candidate target) (84)	Potential promotion of CVB4 infection and impairment of β-cell insulin output, providing a possible link between enteroviral infection and T1D-related β-cell dysfunction. *Antagomir-mediated inhibition reduced infection by both CVB4 strains and modestly increased high-glucose insulin levels after CVB4-E2 infection; downstream targets/pathways were predicted and not functionally validated.*
Vitamin D supplementation/1,25(OH)_2_D treatment	miR-155 (92, 94), arm not specified	Human adipocytes (*in vitro*) & mouse adipose tissue *in vitro* and vivo (92), murine macrophages (RAW264.7, bone marrow-derived macrophages -BMDMs) and human PBMCs (94)	Human adipocytes: n = 3 independent cultures; mice: n = 10 per group (92) RAW264.7 cells: n = 3; BMDMs: n = 3; human PBMC donor number not reported (94)	D	Downregulation	NF-κB signaling (92, 94); miR-155–SOCS1 negative-feedback axis (94)	Potential attenuation of pro-inflammatory signaling relevant to autoimmune β-cell injury. *The effect was demonstrated in adipocyte and macrophage systems; whether vitamin D modulates the miR-155–SOCS1 axis in pancreatic islets or in individuals with T1D remains untested.*
Omega-3 supplementation	miR-34a (97), arm not specified	Mouse liver *in vivo*; human HepG2 cells *in vitro*	Mice: HFD (high fat diet) n = 9 per group (control, HFD and three omega-3 treated HFD groups); HepG2 n= 3	D	Downregulation	SIRT1; downstream SREBP-1c, PGC-1α and CPT-1A (97)	Potential support of β-cell survival through miR-34a/SIRT1-associated signaling. *However, the exposure study primarily demonstrated effects on hepatic lipid metabolism, and whether omega-3 modifies miR-34a in pancreatic islets or in autoimmune T1D has not been tested.*
Single high-fat, high-saturated fat meal	miR-375 (101), arm not specified	Human plasma, *in vivo*	n = 11 healthy women	C	Upregulation	β-cell metabolic signaling and GSIS (72) (mechanistic islet study)	Potential perturbation of β-cell metabolic signaling and glucose-stimulated insulin secretion *The increase in miR-375 was demonstrated in plasma after a single high-fat, high-saturated-fat meal, whereas its effects on β-cell metabolic signaling and GSIS were demonstrated in β-cell/islet models; pancreatic miR-375 and β-cell function were not assessed after meal intake.*
Quercetin	miR-146a (105), arm not specified	Human breast cancer cell lines MCF-7 and MDA-MB-231; orthotopic xenograft in athymic nude mice	Cell lines (replicates not reported); xenograft n = 6 per group	D	Upregulation	EGFR; apoptosis/proliferation/invasion pathways (105) IRAK1/TRAF6–NF-κB relevance is based on independent miR-146a literature (52)	Potential enhancement of miR-146a-mediated restraint of inflammatory signaling relevant to T1D. *Quercetin-induced miR-146a upregulation was demonstrated in breast cancer models, where the functional effects involved EGFR, proliferation, invasion and apoptosis; modulation of the IRAK1/TRAF6–NF-κB axis after quercetin exposure has not been demonstrated in T1D-relevant tissues.*
Epigallocatechin-3-gallate (EGCG)	miR-100-5p (106)	IL-1β-stimulated human osteoarthritis chondrocytes, *in vitro*	Not reported	D	Upregulation	Not functionally assessed in the EGCG exposure study	Potential relevance to T1D is suggested by the opposite direction of miR-100-5p dysregulation reported in T1D-related human samples; the functional significance of this cross-context overlap remains untested.
	let-7a-5p let-7c (106)	IL-1β-stimulated human osteoarthritis chondrocytes, *in vitro*	Not reported	D	Upregulation	Eomes; CD8^+^ T-cell proliferation and CTL differentiation (let-7 family) (107) (murine T-cell study)	Potential attenuation of autoreactive CD8^+^ T-cell differentiation and cytotoxic function, potentially limiting β-cell destruction *EGCG-induced let-7 upregulation was demonstrated in IL-1β-stimulated chondrocytes, whereas the effects on CD8^+^ T-cell differentiation were established for the let-7 family in murine T cells; neither EGCG-mediated modulation of let-7 in T cells nor effects on T1D were directly assessed.*
Extra virgin olive oil with high polyphenol content (H-EVOO)	miR-21-5p (108)	Human plasma; acute postprandial crossover study	n = 12 participants; H-EVOO microRNA analysis n = 10	C	Downregulation	BCL2 (62); *Tgfb2, Smad2* (63)	Potential attenuation of β-cell apoptosis and preservation of β-cell identity and glucose-stimulated insulin secretion *miR-21-5p reduction was observed acutely in plasma after H-EVOO intake in healthy individuals; the tissue of origin and target tissue of the circulating microRNA were unknown, and effects on pancreatic islets or T1D were not assessed.*
Human breast milk/breastfeeding exposure	miR-148a-3p (112)	Human breast milk	n= 16 lactating women	C	Abundance data only: decreased from transition to mature milk but remained markedly higher than in infant formula	DNMT1/FOXP3-associated regulation (115, 116), CaMKIIα (117), IKBKB/IKKβ–NF-κB (118)	Potential support of Treg-mediated immune tolerance and attenuation of pro-inflammatory signaling *miR-148a-3p was measured in maternal milk, not in infants. Uptake and functional effects in infant immune/pancreatic tissues and any impact on T1D risk remain unproven.*
Early-life antibiotic-induced gut dysbiosis (tylosin)	miR-155 (121), arm not specified	Ileum of male NOD mice, *in vivo*	n = 4/group for ileal miRNA analysis	B	Upregulation	TLR2 (relationship based on prior studies cited in (121))	Potential alteration of TLR2-related intestinal immune signaling accompanying enhanced T1D development. *Antibiotic-induced dysbiosis altered ileal miRNA expression and intestinal immune markers; the miRNA–target relationship was not directly validated in this experiment, and miRNA mediation of the T1D effect was not established.*
	miR-145 (121), arm not specified	Ileum of male NOD mice, *in vivo*	n = 4/group for ileal miRNA analysis	B	Upregulation	CD44 (relationship based on prior studies cited in (121))	Potential disruption of intestinal immune signaling associated with enhanced T1D development *Antibiotic-induced dysbiosis altered ileal microRNA profiles together with TLR2 expression, CD44 splicing and Reg3γ regulation, and these changes were partially or fully restored by maternal cecal microbiota transfer. The indicated miRNA–target relationships are based on prior studies and were not directly validated here; whether the microRNA changes causally mediate the observed protection from T1D remains unresolved.*
	miR-143 (121), arm not specified	Ileum of male NOD mice, *in vivo*	n = 4/group for ileal miRNA analysis	B	Upregulation	CD44 and TLR2 (relationships based on prior studies cited in (121))	Potential disruption of the CD44–TLR2 intestinal innate immune network associated with enhanced T1D development *Antibiotic-induced dysbiosis altered ileal microRNA profiles together with TLR2 expression, CD44 splicing and Reg3γ regulation, and these changes were partially or fully restored by maternal cecal microbiota transfer. The indicated miRNA–target relationships are based on prior studies and were not directly validated here; whether the microRNA changes causally mediate the observed protection from T1D remains unresolved.*
	miR-23b (121), arm not specified	Ileum of male NOD mice, *in vivo*	n = 4/group for ileal miRNA analysis	B	Downregulation	Reg3γ-associated regulation (relationship based on prior studies cited in (121))	Potential impairment of intestinal antimicrobial defense and immune regulation accompanying enhanced T1D development. *The miRNA–Reg3γ relationship was not directly validated in this experiment, and causal mediation of the T1D phenotype was not established.*
Gut dysbiosis with ethanolamine accumulation	miR-101a-3p (122)	Mouse ileum and colon, *in vivo*; mouse enteroids and human Caco-2 intestinal epithelial cells, *in vitro*	Mice: n = 5–8/group; cell experiments: 2–3 independent experiments, each performed in triplicate	D	Upregulation	Zo1 mRNA/ZO-1 tight-junction pathway (122)	Potential promotion of intestinal barrier dysfunction and inflammatory signaling relevant to islet autoimmunity. *In obesity/T2D-related intestinal models, miR-101a-3p reduced Zo1 mRNA stability and impaired barrier integrity; this mechanism has not been examined in T1D or islet autoimmunity.*
Air pollution (PM2.5)	miR-146a-5p (127)	Human peripheral blood, *in vivo*	n = 55 healthy young adults	C	Downregulation	TLR2 – predicted target (127), IRAK1/TRAF6–NF-κB regulation is supported by independent miR-146a literature (52)	Potential enhancement of pro-inflammatory immune signaling *PM2.5 exposure was associated with lower blood miR-146a-5p, but direct target validation and T1D-specific outcomes were not assessed. The overlap with T1D therefore represents a candidate rather than established molecular link.* *The direction of change differs from the miR-146a-5p upregulation reported in enteroviral models, suggesting context-dependent regulation across different exposures and tissues; see Section 7 for discussion.*
	miR-155-5p (128)	Human bronchial epithelial cells (BEAS-2B), epithelial-derived exosomes and THP-1-derived macrophages, *in vitro*	≥3 independent assays	D	Upregulation	SOCS1; NF-κB signaling (128)	Potential amplification of pro-inflammatory macrophage signaling relevant to autoimmune β-cell injury. *Exosomal transfer was associated with reduced SOCS1, NF-κB activation and increased inflammatory cytokines in a pulmonary epithelial–macrophage model; whether this pathway occurs systemically or contributes to islet autoimmunity remains unknown.*

Evidence category: A — direct measurement of an environmental factor–microRNA association in individuals with T1D or established islet autoimmunity. B — T1D-relevant experimental system (NOD mice in vivo; human islets or β-cells under a T1D-relevant exposure or stimulus). C — human in vivo exposure or exposure-matrix study with a non-T1D endpoint. D — non-T1D or otherwise non-T1D-specific tissue/disease system. Distribution: A = 0 · B = 7 · C = 10 · D = 11 (28 rows).

Evidence category refers specifically to the evidence supporting the environmental factor–microRNA association; mechanistic evidence for downstream targets or T1D-related consequences may derive from other studies and does not alter the assigned category.

MicroRNA names are reported as they appear in the original publications; where the source did not designate a 5p or 3p arm, none has been inferred. Predicted/in silico targets are labelled as such. Sample sizes refer to biological samples or participants where reported; technical replicates are identified separately when relevant.

Numbers in brackets indicate the corresponding reference(s) supporting the exposure–microRNA association or the downstream target/pathway, as applicable.

**Figure 1:**
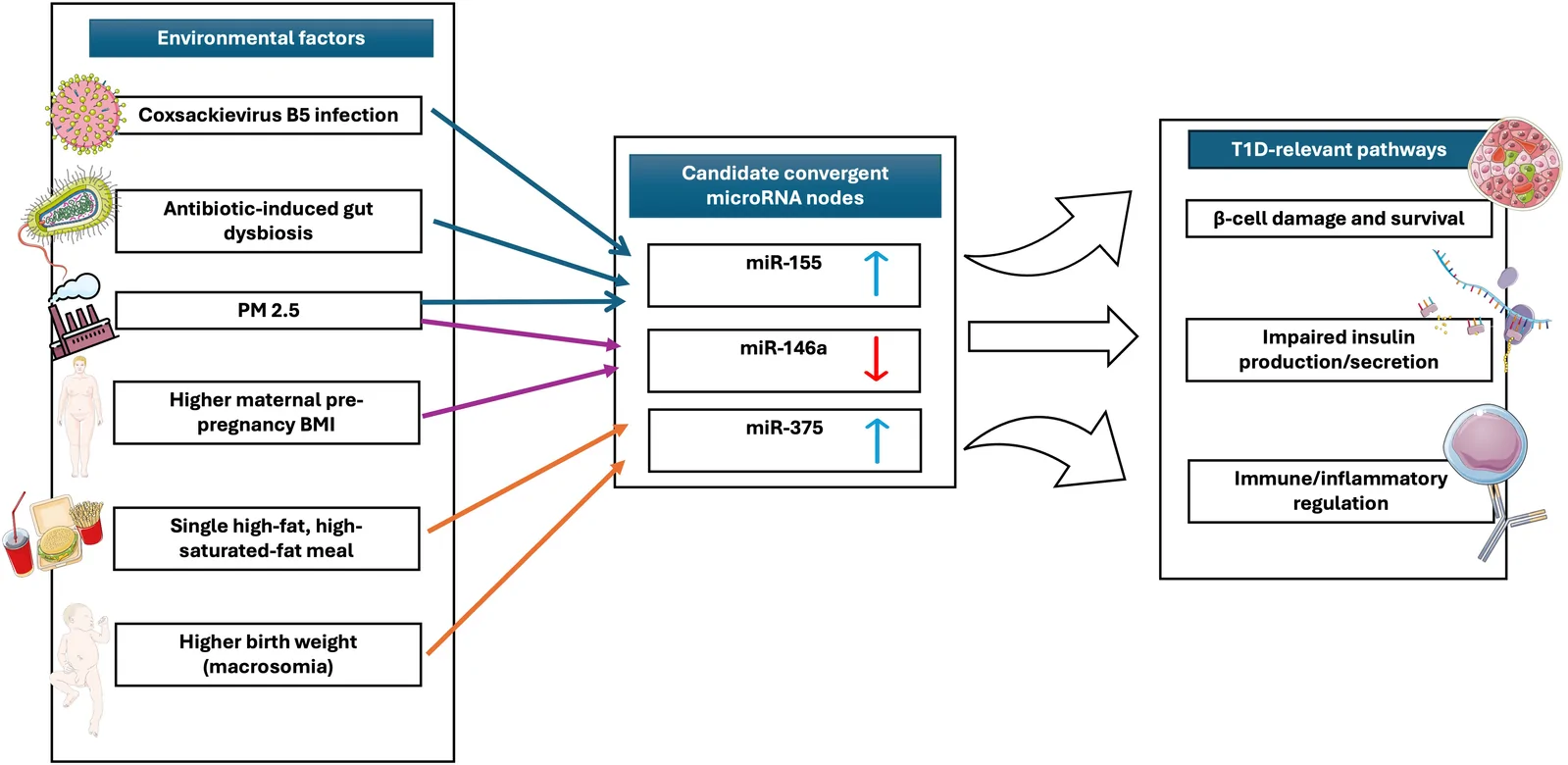
Convergence of selected environmental exposures on recurrent microRNAs relevant to T1D. Selected environmental exposures are associated with recurrent dysregulation of miR-155, miR-146a and miR-375, identifying these microRNAs as candidate convergent regulatory nodes linking environmental influences with T1D-relevant pathways. The figure highlights associations showing concordant directions of microRNA dysregulation across different exposures. Evidence derives from different tissues, populations and experimental models; therefore, the depicted relationships indicate biological convergence rather than an established shared causal mechanism. The association between higher maternal pre-pregnancy BMI and reduced miR-146a expression was observed in female placentas and was modified by gestational weight gain. ↑, upregulation; ↓, downregulation. For graphical consistency, microRNA names in the figure are shown without 5p/3p arm designations; source-specific nomenclature is preserved in the main text and **Table 1**. Where the original study did not specify the mature 5p/3p strand, concordance at the arm level across exposures cannot be established. #Images provided by Servier Medical Art (https://smart.servier.com), licensed under CC BY 4.0 (https://creativecommons.org/licenses/by/4.0/).

### 5.1 Early-life factors

A range of prenatal and early postnatal factors have been associated with subsequent T1D risk (47). Proposed mechanisms include alterations in early immune development and gut microbiota composition, although the pathways linking individual exposures to islet autoimmunity and T1D remain incompletely understood (48).

The human placenta actively participates in maternal–fetal communication by releasing extracellular vesicles into both the maternal and fetal circulations. These vesicles carry bioactive molecules, including microRNAs, and may contribute to signaling between the placenta and fetal tissues (49). This provides a plausible route through which changes in the maternal environment could influence fetal development by alterations in placental signaling, including microRNA-containing extracellular vesicles, although the relevance of this pathway to subsequent T1D risk remains unknown.

Among prenatal factors associated with subsequent T1D risk are greater maternal weight gain during pregnancy and gestational hypertension (48).

Maternal adiposity has been linked to altered placental microRNA profiles, with several microRNAs downregulated in placentas from women with pregestational overweight/obesity or gestational obesity (50).

In the ENVIRONAGE Birth Cohort, higher maternal pre-pregnancy body mass index (BMI) was associated with lower placental miR-146a expression in female offspring, although this association depended on gestational weight gain and was not observed in males (51).

miR-146a functions as a negative-feedback regulator of inflammatory signaling: NF-κB (nuclear factor kappa B)-dependent induction of miR-146a suppresses TRAF6 (TNF receptor-associated factor 6) and IRAK1 (IL-1 receptor-associated kinase 1), thereby limiting excessive inflammatory responses (52). Reduced miR-146a expression has also been reported in T1D. Yang et al. found a 48% reduction in miR-146a expression in peripheral blood mononuclear cells (PBMCs) from individuals with newly diagnosed T1D compared with healthy controls, with lower expression also associated with higher glutamic acid decarboxylase autoantibodies (GADA) levels (53). Similarly, serum miR-146a-5p levels were lower in patients with T1D than in those with type 2 diabetes (T2D) and nondiabetic controls, whereas the reduction observed in latent autoimmune diabetes in adults (LADA) did not reach statistical significance (54).

Reduced miR-146a expression may therefore weaken negative feedback on NF-κB signaling, potentially enhancing inflammatory signaling and contributing to endoplasmic reticulum and oxidative stress and β-cell apoptosis (21).

However, these observations derive from distinct populations and biological compartments and therefore do not establish a continuous mechanistic pathway linking maternal adiposity to T1D.

Moreover, circulating exosomal miR-210-3p expression was higher in women with gestational hypertension and preeclampsia than in normotensive pregnant women, with the highest levels observed in severe preeclampsia (55). Although placental extracellular vesicles are released into the fetal circulation (49), whether altered maternal or placental miR-210 levels are reflected in the fetus has not been established. Assmann et al. found elevated plasma miR-210-3p in patients within the five years of T1D diagnosis (56), whereas Osipova et al. reported increased miR-210 levels in both plasma and urine of children with T1D (57). Another study showed that miR-210 directly suppresses FOXP3, which is essential for regulatory T-cell function (58). This mechanism, however, has not been examined directly in T1D.

A nationwide cohort study found that maternal autoimmune disease was associated with a higher risk of childhood-onset T1D in offspring, including Hashimoto’s thyroiditis and inflammatory bowel disease (59). MicroRNA dysregulation could represent one of several biological links between maternal autoimmunity and offspring risk. However, the studies discussed below assessed microRNAs in affected adults, rather than during pregnancy or in offspring. Whether similar alterations occur during pregnancy or are present in the fetus therefore remains unclear.

Patients with Hashimoto’s thyroiditis had higher serum miR-21-5p expression than healthy controls (60). Altered miR-21-5p expression has also been reported in T1D, although the direction of change differs between studies and sample types (54, 61). Overexpression of miR-21-5p promoted β-cell apoptosis through direct targeting of *BCL2* mRNA, resulting in reduced BCL2 protein levels and increased cleaved caspase-3 in rat β-cells and human islets (62). Another study showed that β-cell pre-miR-21 induction impaired glucose-stimulated insulin secretion and promoted loss of β-cell identity, with miR-21-5p directly targeting *Tgfb2* and *Smad2*, components of TGF-β (transforming growth factor beta) signaling involved in maintaining β-cell identity and differentiation (63).

Inflammatory bowel disease is associated with altered microRNA expression, with miR-21-5p consistently upregulated in both ulcerative colitis and Crohn’s disease, whereas miR-155-5p appears to be more consistently increased in ulcerative colitis (64). In T1D, Assmann et al. observed a trend toward higher plasma miR-155-5p levels in patients within five years of diagnosis, although the difference from healthy controls did not reach statistical significance in the validation cohort (56). More recently, exposure of human pancreatic islets to IL-1β and IFN-γ (interferon gamma) increased miR-155-5p in both islets and islet-derived extracellular vesicles, together with miR-146a-5p (65). miR-155-5p was also significantly elevated in plasma-derived extracellular vesicles from individuals with recent-onset T1D compared with healthy controls (65).

T-cell-derived exosomes containing miR-155 were shown to transfer their microRNA cargo to pancreatic β-cells, resulting in NF-κB activation, increased expression of the pro-inflammatory chemokines CCL2, CCL7 and CXCL10, and β-cell apoptosis (35). Overexpression of miR-155 alone was sufficient to promote β-cell apoptosis and NF-κB activation, supporting its contribution to these effects (35). In NOD mice, β-cell-specific inhibition of several microRNAs, including miR-155, using a miRNA sponge reduced diabetes incidence from 80% to 40% and was associated with lower insulitis scores (35). In diabetic kidney disease, miR-155-5p suppressed SOCS1 and promoted STAT1/3 activation, accompanied by increased expression of CCL2, CXCL10, TNF-α (tumor necrosis factor alpha) and IL-6 (66). This provides an additional mechanism by which miR-155-5p could amplify inflammatory signaling, although this pathway has not been examined directly in pancreatic β-cells and in the context of T1D.

Findings also suggest that higher birth weight (48, 67) and greater birth length are associated with an increased risk of T1D later in life (48). In newborns with different birth weights, miR-375 expression in dried blood spots was 1.7-fold higher in the macrosomic group than in the normal-birth-weight group (68). miR-375 is highly expressed in pancreatic β-cells and is involved in the regulation of β-cell development, proliferation, survival and insulin secretion (69). In porcine pancreatic stem cells, miR-375 overexpression promoted apoptosis and inhibited proliferation and differentiation into islet-like cells, while suppressing the PDK1–AKT signaling pathway (70); PDK1 was identified as a direct target of miR-375 in this model (70). In rat INS-1 β-cells, miR-375 directly targeted *Psen1*, which encodes presenilin 1, a catalytic component of the γ-secretase complex, thereby reducing PSEN1 protein and the levels of NICD1, the active intracellular domain of Notch1 involved in regulating cell fate and survival (71). miR-375 overexpression was also associated with increased β-cell apoptosis, although this mechanism was studied in the context of glucotoxicity and together with other microRNAs, limiting its direct applicability to human T1D (71).

Evidence from primary rat and human islets indicates that elevated miR-375 impairs glucose-stimulated insulin secretion (GSIS), accompanied by altered expression of genes involved in β-cell glucose metabolism, including *Pc*, *Pdk4*, and *Mdh1* (72). Although these experiments were not performed in a T1D setting, impaired β-cell glucose metabolism and insulin secretion could also be relevant to β-cell dysfunction in T1D.

Taken together, the available evidence suggests that early-life exposures, particularly maternal conditions and birth-related factors, are associated with alterations in microRNAs with potential links to β-cell function and T1D pathogenesis. However, it remains unclear whether maternal microRNA alterations are transmitted to the fetus or whether they mediate the association between early-life exposures and subsequent T1D risk.

### 5.2 Infectious agents

Viral infections during early childhood and *in utero* have long been investigated as potential triggers of islet autoimmunity and progression to T1D (73). Seasonal variation in T1D incidence, with lower incidence during the summer months, has also been observed and broadly parallels the seasonal pattern of many viral infections (6). In a recent systematic review and meta-analysis, Sung et al. found that childhood respiratory tract infections were associated with an increased risk of T1D (odds ratio (OR) 1.47, 95% confidence interval (CI) 1.23–1.75), whereas no significant association was observed with islet autoimmunity (74). However, the included studies were observational and showed substantial heterogeneity, limiting causal interpretation.

While many viruses have been associated with T1D, enteroviruses (EVs) have been studied most extensively and are considered the leading viral candidates implicated in islet autoimmunity and T1D (73, 75).

Kim et al. profiled microRNA expression in Coxsackievirus B5 (CVB5)-infected human pancreatic islets and identified 33 significantly dysregulated microRNAs. Among them, miR-155-5p increased fourfold, whereas miR-34a-5p showed the greatest increase, reaching approximately eightfold (76). GLIS3, a T1D susceptibility gene involved in β-cell survival, was identified as a predicted target of miR-155-5p. However, GLIS3 mRNA expression was not significantly altered in the infected islets, and direct regulation by miR-155-5p was not validated in this model (76).

In another study using MIN6 cells, miR-34a overexpression reduced glucose-stimulated insulin secretion and VAMP2 expression and increased β-cell apoptosis, accompanied by reduced levels of the anti-apoptotic protein BCL2 (77). These effects were not directly examined in CVB5-infected human islets.

Interestingly, the effects of miR-34a may be context-dependent. In NOD.B10 Idd9.3 mice, higher miR-34a expression in B-cell progenitors was associated with reduced Foxp1 expression, impaired B-cell lymphopoiesis and less efficient priming of islet-specific CD4^+^ T cells, changes that were linked to protection from T1D (78).

Persistent Coxsackievirus B4 (CVB4) infection of the human pancreatic ductal PANC-1 cell line dysregulated 81 microRNAs, including an 8.5-fold increase in miR-204-5p and a 5.4-fold increase in miR-146a-5p (79). It was also demonstrated that miR-204 directly targeted *MafA*, a transcription factor important for insulin gene expression, leading to reduced insulin production in β-cells; this effect was also observed in primary human islets (80). Increased miR-146a may also affect antiviral responses. Ho et al. showed that enterovirus 71-induced miR-146a directly suppressed IRAK1 and TRAF6, key components of Toll-like receptor signaling involved in type I interferon production (81). Inhibition of miR-146a restored IRAK1 and TRAF6 expression, increased IFN-β production and restricted viral propagation (81). The relevance of miR-146a to β-cell injury is further supported by Roggli et al., who found that pro-inflammatory cytokines increased miR-146a expression in MIN6 cells and human pancreatic islets, while miR-146a overexpression promoted apoptosis and its inhibition partially protected MIN6 cells from cytokine-induced cell death (77). Several let-7 3p species, including let-7f-1-3p, let-7b-3p and let-7d-3p, were also downregulated after persistent CVB4 infection (79). Although the let-7 family has been implicated in glucose homeostasis and insulin sensitivity in experimental models (82), the functional consequences of these specific 3p alterations were not assessed in CVB4-infected cells. miR-10a-5p was likewise downregulated after persistent CVB4 infection (79). Its mouse homolog has previously been reported at lower levels in regulatory T cells from diabetes-prone NOD mice than in autoimmunity-resistant mice (83), suggesting a possible link with autoimmune regulation. However, these downstream mechanisms were not directly examined in CVB4-infected PANC-1 cells, and the relevance of these findings to pancreatic β-cells remains uncertain.

In line with these observations, Lalani et al. identified hsa-miR-AMC1, a novel microRNA, as upregulated after CVB4 infection in the human pancreatic β-cell line EndoC-βH1 (84). Inhibition of hsa-miR-AMC1 with an antagomir significantly reduced infection by both the reference CVB4-JVB and the diabetogenic CVB4-E2 strains (84). In cells infected with CVB4-E2, hsa-miR-AMC1 inhibition also produced a modest increase in glucose-stimulated insulin levels, whereas this effect was not observed with the JVB strain (84). Bioinformatic analysis identified 64 predicted hsa-miR-AMC1 target genes enriched in the insulin/IGF (insulin-like growth factor)-MAPK (mitogen-activated protein kinase) signaling pathway; 13 predicted targets were also differentially expressed in β-cells infected with CVB4-E2 (84). However, these targets were not functionally validated, and the molecular mechanism through which hsa-miR-AMC1 affects viral infection and β-cell function remains unclear. These findings suggest that CVB4-induced hsa-miR-AMC1 may promote CVB4 infection and contribute to impaired β-cell function, providing a potential link between enteroviral infection and T1D pathogenesis.

Because enterovirus infections are common and frequently asymptomatic, identifying individuals at increased risk remains challenging (85, 86). Preventive vaccines targeting selected enteroviruses are therefore being investigated as a potential strategy to reduce the risk of T1D (87).

### 5.3 Dietary factors

The role of diet in the pathogenesis of T1D has been the subject of extensive investigation, with a growing body of evidence suggesting a role for several dietary factors (88). Risk of islet autoimmunity or T1D was positively associated with childhood consumption of cow’s milk, sugar, sugar-sweetened beverages, carbohydrates, protein, nitrites and meat. On the other hand, inverse associations have been observed for breastfeeding, delayed exposure to cow’s milk and gluten, fruit consumption, vitamin D supplementation in infancy, higher 25(OH)D concentrations in adulthood, and childhood consumption of vitamins A and C (88). Together, these observations indicate that diet may contribute to T1D risk; however, the evidence is stronger for some exposures than for others.

Some dietary factors have also been shown to modulate the expression of microRNAs that are involved in pathways relevant to T1D pathogenesis.

Vitamin D deficiency has been associated with an increased risk of T1D in early life, particularly in children who are genetically susceptible (89). Vitamin D exerts several immunomodulatory effects through the vitamin D receptor (VDR), a nuclear transcription factor involved in the regulation of genes controlling immune and inflammatory responses (90). The higher incidence of T1D observed in regions with lower sunlight exposure such as Finland and Norway has also been discussed as indirect support for a possible role of vitamin D in disease pathogenesis (91).

Vitamin D has been shown to modulate the expression of inflammation-associated miR-155. In human adipocytes, 1,25(OH)_2_D _(_active form of vitamin D) attenuated TNF-α-induced miR-155 expression, while vitamin D supplementation reduced miR-155 expression in adipose tissue of high-fat-diet-fed mice. Further experiments in murine adipocytes showed that 1,25(OH)_2_D inhibited NF-κB activation, a pathway involved in miR-155 regulation (92).

This effect was also observed in murine macrophage models, where 1,25(OH)_2_D suppressed lipopolysaccharide (LPS)-induced miR-155 expression and increased SOCS1, a negative regulator of inflammatory signaling targeted by miR-155 (93, 94). These findings support a vitamin D-miR-155-SOCS1 regulatory axis in macrophages. However, whether vitamin D supplementation modulates this pathway in individuals with T1D or in pancreatic islets remains unknown.

The role of omega-3 polyunsaturated fatty acids in the prevention of type 1 diabetes remains uncertain, with inconsistent findings across studies (95, 96). In high-fat-diet-fed mice and palmitate-treated HepG2 cells, omega-3 fatty acids reduced miR-34a and SREBP-1c (sterol regulatory element-binding protein-1c) expression while increasing SIRT1 (sirtuin 1), PGC-1α and CPT-1A, a pattern consistent with reduced lipogenesis and enhanced fatty acid oxidation, and accompanied by lower hepatic lipid accumulation (97). In the same study, miR-34a overexpression reduced SIRT1 expression and attenuated the metabolic effects of eicosapentaenoic acid (EPA) in HepG2 cells (97). SIRT1 is a NAD^+^-dependent deacetylase that promotes insulin secretion, counteracts pro-inflammatory signaling, and protects pancreatic β-cells from apoptosis (98). Increased circulating miR-34a has been reported in individuals with T1D (99), while miR-34a-mediated suppression of Bcl-2 has been shown to promote apoptosis in murine MIN6 β-cells (100). These findings suggest a possible link between omega-3-associated changes in miR-34a and pathways relevant to β-cell survival. However, the effects observed in the omega-3 study were confined to hepatic lipid metabolism and lipid accumulation, and whether omega-3 modulates miR-34a in pancreatic islets or in the context of T1D has not been examined.

It was shown that intake of a single high-fat, high-saturated-fat meal increased circulating miR-375 in healthy women, with significant elevations observed 3 and 5 hours after meal (101). This finding is notable because miR-375 is involved in β-cell metabolic signaling and glucose-stimulated insulin secretion, as discussed above (72). It suggests that postprandial lipid exposure may engage microRNA pathways relevant to β-cell function.

In a complementary animal model, miR-34a-5p was the most significantly upregulated of 24 differentially expressed microRNAs in the pancreas of high-fat-diet-fed Zucker diabetic fatty rats (102), although this model reflects type 2 rather than autoimmune diabetes. This direction of change is consistent with the previously discussed evidence linking increased miR-34a expression to reduced SIRT1 expression and suppression of Bcl-2, thereby favoring β-cell apoptosis, although the omega-3 study did not specify the mature miR-34a strand (97). The increase in miR-34a-5p therefore extends this observation to pancreatic tissue and raises the possibility that lipid-related metabolic conditions may converge on miR-34a-associated pathways relevant to β-cell dysfunction. However, because diet, genotype and diabetic phenotype differed between groups, the contribution of high-fat feeding itself cannot be isolated.

Owing to their antioxidant, anti-inflammatory, and immunomodulatory properties, polyphenols - naturally occurring bioactive compounds widely distributed in plant-derived foods— have attracted considerable interest as potential adjunctive approaches in T1D (103). Several polyphenols may influence pathways relevant to T1D by reducing oxidative stress, modulating immune responses, preserving pancreatic β-cell function, and enhancing insulin secretion, thereby potentially contributing to improved glucose homeostasis (103). However, most of the supporting evidence derives from *in vitro* and animal models, and clinical evidence in individuals with T1D remains limited (103).

Experimental studies have also shown that polyphenols may regulate microRNA expression and thereby influence post-transcriptional gene expression (104).

Some of the microRNAs dysregulated in T1D overlap with those shown to be modulated by dietary polyphenols.

Quercetin, abundant in such products as apples, grapes, red onions and black and green tea (104), has been demonstrated to upregulate miR-146a in human breast cancer cells (105). Given the role of miR-146a in limiting NF-κB signaling, this finding suggests that quercetin may affect inflammatory pathways relevant to T1D through miR-146a. However, whether a similar effect occurs in immune cells or pancreatic islets has not been examined.

Similarly, epigallocatechin-3-gallate (EGCG), a polyphenol abundant in green tea (104), has been shown to upregulate miR-100-5p in IL-1β-stimulated human osteoarthritis chondrocytes (106). Reduced miR-100-5p expression has also been reported in T1D-related human samples (61). Although these findings come from different biological settings, the opposite direction of change suggests that miR-100-5p may be one of the diet-responsive microRNAs relevant to T1D. Whether EGCG alters miR-100-5p expression in immune cells or pancreatic tissue is unknown.

Furthermore, EGCG upregulates multiple members of the let-7 family — including let-7a-5p and let-7c in IL-1β-stimulated human chondrocytes (106). Both microRNAs have been reported to be downregulated in pancreatic tissue from diabetic mice and in PBMCs/T cells from individuals with T1D (61).

Wells et al. showed that high let-7 expression maintains the naive state of murine CD8^+^ T cells, whereas T-cell receptor-mediated downregulation of let-7 is required for their differentiation into cytotoxic T lymphocytes (CTLs) (107). Forced let-7 expression impaired CD8^+^ T-cell proliferation, differentiation and cytotoxic function, partly through suppression of the transcription factor Eomes (107). Given the role of cytotoxic T cells in pancreatic β-cell destruction, these findings suggest that maintaining higher let-7 expression in autoreactive CD8^+^ T cells could potentially limit their effector response. However, the EGCG-induced increases in let-7a-5p and let-7c described above were observed in human chondrocytes, limiting the direct relevance of these findings to T-cell regulation in T1D.

Collectively, these observations support the possibility that specific polyphenols may influence microRNA pathways relevant to T1D. However, the polyphenol–microRNA interactions described above were characterized mainly in cancer and inflammatory cell models, and have not been demonstrated in T1D-relevant immune or pancreatic tissues.

Acute intake of high-polyphenol extra virgin olive oil (H-EVOO) reduced circulating miR-21-5p levels in healthy volunteers (108). miR-21-5p dysregulation has also been reported in T1D, although its direction varies across studies and sample types (54, 61), while studies in rodent and human islets have linked miR-21-5p to β-cell apoptosis through BCL2 suppression and to impaired β-cell function and identity through targeting of Tgfb2 and Smad2 (62, 63). The relevance of the H-EVOO-associated reduction in circulating miR-21-5p to pancreatic islet biology or T1D therefore remains uncertain.

Another dietary factor that may influence T1D risk is breastfeeding. In a large prospective cohort study involving more than 155,000 children from Norway and Denmark, absence of breastfeeding was associated with an approximately twofold higher risk of T1D compared with those who received breast milk (109). However, among breastfed children, neither the duration of exclusive breastfeeding nor the total duration of breastfeeding was associated with disease risk (109). Similarly, in the Environmental Determinants of Diabetes in the Young (TEDDY) study, longer breastfeeding duration was not associated with a reduced risk of islet autoimmunity in genetically susceptible children (110).

Human breast milk contains abundant microRNAs, including miR-148a-3p (111). In the study by Chiba et al. miR-148a-3p levels decreased from transition to mature milk but remained markedly higher in mature breast milk than in infant formula (112). Human milk exosomes and their microRNA cargo have been shown to survive simulated gastrointestinal digestion *in vitro* and to be taken up by human intestinal cells (113, 114), although the extent to which dietary microRNAs are absorbed and remain functionally active in human infants is uncertain.

Studies have linked miR-148a to DNMT1 (DNA methyltransferase 1) regulation and DNMT1-dependent control of FOXP3 locus methylation and inducible regulatory T-cell differentiation (115, 116). Members of the miR-148/152 family have also been shown to regulate CaMKIIα (calcium/calmodulin-dependent protein kinase IIα), a regulator of dendritic cell maturation and function, thereby reducing pro-inflammatory cytokine production and antigen-presenting activity (117), while miR-148a-3p directly represses IKBKB, which encodes the NF-κB regulator IKKβ, thus attenuating NF-κB signaling and inflammatory gene expression in human aortic valve cells (118). These pathways provide a possible immune-regulatory context in which breast milk miR-148a-3p could be relevant to T1D. However, these mechanisms have not been demonstrated following exposure to breast milk miR-148a-3p in infants or in T1D-specific models.

Thus, breast milk provides a dietary source of miR-148a-3p with potential immune-regulatory relevance, but whether milk-derived miR-148a-3p contributes to the association between breastfeeding and T1D risk remains unknown.

Overall, dietary exposures can modulate microRNAs that interact with pathways implicated in T1D, including β-cell survival, inflammatory signaling and immune regulation. These observations provide a basis for considering microRNAs as candidate molecular links between nutrition and T1D-related biology, although direct evidence that diet-induced microRNA changes modify T1D risk in humans is limited.

### 5.4 Gut microbiota

Over the past decade, advances in sequencing technologies and data analysis have substantially accelerated research on the gut microbiome (119). Gut microbiota dysbiosis has been associated with T1D, with altered intestinal permeability and immune regulation proposed as potential mechanisms linking microbial changes to disease development (120). Increased intestinal permeability may facilitate the passage of microbial antigens and products across the gut barrier, potentially promoting intestinal inflammation and activation of immune responses relevant to islet autoimmunity (120). Reduced abundance of butyrate-producing bacteria has also been reported in children with islet autoimmunity and in individuals with T1D, while butyrate contributes to intestinal epithelial barrier function and immunological regulation (120). However, the mechanisms linking these microbiota alterations to T1D remain incompletely understood. Evidence directly connecting microbiota-induced changes in microRNA expression with T1D is particularly limited.

Zhang et al. showed that early-life treatment with the macrolide antibiotic tylosin in male NOD mice disrupted the gut microbiota, depleting several commensal taxa, including members of the S24-7 family and the genera *Oscillospira* and *Dorea*, while enriching members of the Enterobacteriaceae family (121). Notably, lower early-life abundance of *Oscillospira* in untreated mice was associated with earlier T1D development, leading the authors to propose its depletion as a potential early marker of disease onset (121). Antibiotic-induced dysbiosis was also accompanied by changes in ileal microRNA expression. miR-145, miR-143 and miR-155, which had previously been reported to regulate CD44 and/or TLR2 (Toll-like receptor 2), were increased, whereas miR-23b, previously linked to regulation of Reg3γ (regenerating islet-derived protein 3 gamma), was decreased (121). Maternal cecal microbiota transfer (CMT) largely restored both the microbial composition and these microRNA expression patterns and also reversed much of the antibiotic-associated increase in T1D incidence; at 30 weeks, 50% of control mice remained T1D-free compared with 15% of antibiotic-treated mice and 45% of mice receiving CMT (121). These findings provide experimental evidence that early-life microbiota perturbation can influence T1D development and intestinal microRNA expression, although they do not establish that the observed microRNA changes mediate the effect on diabetes development.

A potential microbiota–microRNA mechanism relevant to intestinal barrier function has been described outside the T1D setting. In obese and diabetic mouse models, mouse intestinal enteroids and human Caco-2 cells, Mishra et al. showed that reduced microbial capacity to metabolize ethanolamine led to its accumulation in the gut and increased intestinal miR-101a-3p expression. Increased miR-101a-3p reduced the stability of *Zo1* (zonula occludens-1) mRNA, weakening the intestinal barrier and promoting increased gut permeability, inflammation and abnormalities in glucose metabolism (122). Although this mechanism has not been demonstrated in T1D, Santos et al. found approximately threefold higher circulating miR-101-3p levels in patients with recent-onset T1D and in normoglycemic individuals with multiple islet autoantibodies compared with controls and individuals with a single autoantibody (123). In silico analysis further identified HGF/c-Met, ephrin receptor and STAT3 signaling among pathways potentially targeted by miR-101-3p and relevant to β-cell function and survival (123). However, the intestinal miR-101a-3p mechanism described by Mishra et al. and the circulating miR-101-3p alterations observed in T1D by Santos et al. were examined in separate disease contexts, and it remains unknown whether microbiota-induced miR-101-3p-mediated barrier dysfunction contributes to islet autoimmunity or T1D development.

### 5.5 Environmental pollutants

Air pollution has been investigated as a potential environmental contributor to T1D, with several epidemiological studies reporting associations between exposure to particulate matter (PM) and T1D incidence or risk (124). PM may also serve as a carrier of xenobiotic toxicants, including heavy metals and organic pollutants, which can increase their distribution and bioavailability (125). Epidemiological studies have examined associations with both PM10 (particles with an aerodynamic diameter ≤10 µm) and PM2.5 (≤2.5 µm), although findings across populations have not been entirely consistent (124).

Environmental air pollutants have been proposed to contribute to T1D through disruption of immune homeostasis, metabolic dysregulation, and direct or indirect injury to pancreatic islets (126). MicroRNA dysregulation may represent one of the epigenetic mechanisms involved in these effects, although direct evidence linking specific pollutants to T1D-related microRNA alterations remains limited.

One potential link between particulate air pollution and T1D involves miR-146a-5p. In a randomized crossover study of 55 healthy young adults, Chen et al. found that short-term exposure to higher PM2.5 levels was associated with reduced peripheral blood miR-146a-5p expression (127). miR-146a-5p, previously discussed as a negative regulator of inflammatory signaling, has also been reported to be downregulated in individuals with T1D (61). The concordant direction of these alterations suggests that miR-146a-5p may represent a molecular link between PM2.5 exposure and immune pathways relevant to T1D. However, Chen et al. studied healthy individuals and did not assess islet autoimmunity or T1D, so whether PM2.5-induced miR-146a-5p changes contribute to disease development remains unknown.

Further *in vitro* evidence indicates that PM2.5 exposure increased miR-155-5p expression in human bronchial epithelial BEAS-2B cells and in their derived exosomes (128). Exposure of THP-1-derived macrophages to exosomes from PM2.5-treated BEAS-2B cells was accompanied by increased miR-155-5p expression, reduced SOCS1 expression, activation of NF-κB and increased IL-1β, IL-6 and TNF-α levels, consistent with involvement of the miR-155-5p/SOCS1/NF-κB axis in the inflammatory response (128). Interestingly, miR-155-5p was also significantly increased in plasma-derived extracellular vesicles from individuals with recent-onset T1D compared with non-diabetic controls (65). This convergence suggests a potential link between PM2.5-responsive miR-155-5p and immune pathways relevant to T1D. However, the PM2.5 study was restricted to an *in vitro* pulmonary epithelial–macrophage model, and it remains unknown whether PM2.5-induced miR-155-5p alterations occur systemically or contribute to islet autoimmunity.

Given widespread exposure to air pollution, further studies are needed to determine whether PM-induced microRNA dysregulation contributes to immune dysfunction involved in T1D pathogenesis.

## 6 Potential clinical relevance of identified microRNA dysregulation in T1D

According to current International Society for Pediatric and Adolescent Diabetes (ISPAD) guidelines, T1D progresses through four stages, from presymptomatic islet autoimmunity to clinically manifest and long-standing disease (129). Stages 1 and 2 are characterized by the presence of multiple islet autoantibodies, with normoglycemia in stage 1 and dysglycemia in stage 2, whereas stage 3 corresponds to clinically manifest T1D and stage 4 to long-standing disease (129). Earlier identification of individuals at risk of progression to clinical T1D is increasingly important because it may reduce the risk of presentation with diabetic ketoacidosis and enable timely consideration of interventions aimed at delaying disease progression, including teplizumab for eligible individuals with stage 2 T1D (129).

A proposed “stage 0” has also been used to describe the period preceding islet autoantibody seroconversion in individuals at increased risk of T1D (7). Research is ongoing to identify biomarkers capable of distinguishing individuals who will subsequently develop islet autoimmunity and T1D, with the aim of improving early risk stratification and prediction (7).

Although islet autoantibodies remain central biomarkers of T1D risk, they indicate established islet autoimmunity rather than directly reflecting ongoing β-cell injury and have limited utility for monitoring the rate of disease progression (130). The considerable heterogeneity in T1D progression therefore supports the need for complementary biomarkers that capture dynamic changes occurring during disease development (130).

Thus, due to their stability and detectability, microRNAs have emerged as promising candidate biomarkers in T1D and may also represent potential therapeutic targets (131).

In a recent study published in *Nature Medicine*, Joglekar et al. developed a dynamic microRNA-based risk score (PREDICT T1D) based on a panel of 50 microRNAs selected from developing human pancreas, human islets and non-pancreatic tissues, plasma from individuals with newly diagnosed T1D and controls, and previously published studies. Plasma microRNA levels and age at sampling were analyzed in 2,204 participants from Australia, Denmark, Hong Kong and India using machine learning (132).

The score was described as dynamic because it incorporated circulating microRNA levels, which can change over time and in response to environmental influences, together with age at sampling; environmental exposures themselves were not measured. To enhance the model, the authors generated synthetic control profiles using generative AI. The model augmented with 100,000 synthetic controls, termed the enhanced four-context dynamic risk score (eDRS4C-100,000), achieved an area under the curve (AUC) of 0.841 in an independent validation cohort of 662 participants from five countries. A single longitudinal case was also presented, in which the score was elevated from the first available sampling at 14 months of age, whereas autoantibodies increased from 39 months. Because no earlier sample was available, this case does not allow estimation of lead time, but it illustrates the longitudinal changes in the score that cannot be captured by cross-sectional measurements (132).

In the TEDDY study, autoantibodies and genetic risk score 2 (GRS2) yielded AUCs of 0.75 and 0.73, respectively (133). However, these estimates concerned prediction of future T1D in genetically at-risk children and are therefore not directly comparable with the PREDICT T1D case-control discrimination of established T1D. In the Danish sibling cohort, genetic risk scores (n = 163) and autoantibody data (n = 168) were available, and the PREDICT T1D signature showed performance comparable to these established biomarkers (132).

The study also explored potential treatment-related applications. In a small subgroup of an imatinib trial (n = 11 in each of the upper and lower quartiles of treatment response), baseline microRNA profiles distinguished individuals with the highest and lowest C-peptide responses at one year, whereas baseline clinical and biochemical parameters did not. In addition, among 31 islet transplant recipients, the eDRS4C-100,000 score predicted insulin requirement one month after transplantation most accurately when measured 1 h after transplantation (AUC 0.820), but less well at 24 h (AUC 0.714) and not before transplantation (AUC 0.444). This pattern is consistent with transient release of microRNAs from stressed or dying β-cells (132).

Notably, seven microRNAs discussed earlier in this review — miR-21-5p, miR-34a-5p, miR-155, miR-375-3p, miR-210-3p, miR-148a-3p and miR-146a-5p — were included in the 50-microRNA PREDICT T1D panel. Their inclusion does not constitute independent validation, as the panel was partly informed by previous literature. It does, however, show that these microRNAs were selected as informative by a model trained across four geographical settings. Among them, miR-155 ranked among the ten variables contributing most to model performance (132).

Overall, the study suggests that circulating microRNAs may provide information relevant to T1D diagnosis, treatment response and the outcome of islet transplantation. The transplantation findings are also relevant to the interpretation of circulating microRNAs: the score was informative shortly after transplantation but not before it, consistent with a signal related to β-cell injury rather than necessarily indicating a causal role. Whether these microRNAs are simply markers of β-cell injury or also contribute to β-cell responses to environmental triggers remains unknown.

Despite significant potential, the clinical use of microRNA-based approaches in T1D remains challenging. The complexity of regulatory networks, in which one microRNA can target multiple genes and individual genes can be regulated by multiple microRNAs, complicates the identification and biological interpretation of disease-specific signatures (131).

Practical implementation is also limited by time-sensitive sample handling. In the context of T1D biomarker studies, miRNA analysis within 8 hours of sample collection has been highlighted as a logistical challenge, particularly in multicenter studies (134). More specifically, best-practice recommendations for circulating microRNA measurements advise processing blood samples within 2 hours of collection to minimize pre-analytical variability (135). Circulating microRNA measurements are further affected by hemolysis and blood-cell contamination, differences in sample collection and processing, RNA extraction and normalization procedures, and analytical methods (135, 136). The influence of hemolysis varies across individual microRNAs; for example, miR-21 has been shown to be susceptible to its effects (137). Analytical variability may additionally arise from differences in detection platforms, including qRT-PCR, next-generation sequencing (NGS) and droplet digital PCR (ddPCR), as well as from the use of different normalization strategies, including exogenous synthetic reference microRNAs, endogenous reference microRNAs and normalization to the average expression of detected microRNAs (138). Such methodological heterogeneity may contribute to poor reproducibility between studies (136, 138). Standardized pre-analytical workflows, consistent analytical and normalization procedures, and further validation in large independent cohorts are therefore needed before circulating microRNA biomarkers can be implemented in routine clinical practice (135, 136).

MicroRNA-based therapies remain an emerging therapeutic approach, as no microRNA-based drug has yet reached phase III trials or received FDA approval (139). MRX34, a synthetic miR-34a mimic, was the first microRNA-based cancer therapy to enter clinical testing, with a phase I trial initiated in 2013. The trial was terminated in 2016 following serious immune-mediated adverse events, including fatal cases (140). More broadly, the pleiotropic nature of microRNA regulation, whereby individual microRNAs can affect multiple targets, may increase the risk of off-target effects and toxicity and represents an important challenge for therapeutic development (139).

Several dietary compounds have been shown to modulate the expression of microRNAs that are also dysregulated in T1D, suggesting that dietary interventions might represent a potential means of influencing disease-relevant microRNA pathways. However, current evidence derives largely from non-T1D experimental models, and it remains unknown whether such interventions can meaningfully alter microRNA expression in pancreatic islets or influence T1D progression in humans.

Nevertheless, despite these limitations, microRNAs remain promising candidates for both diagnostic and therapeutic applications in T1D. The potential clinical applications of environmentally responsive microRNAs are summarized in **Figure 2**.

**Figure 2:**
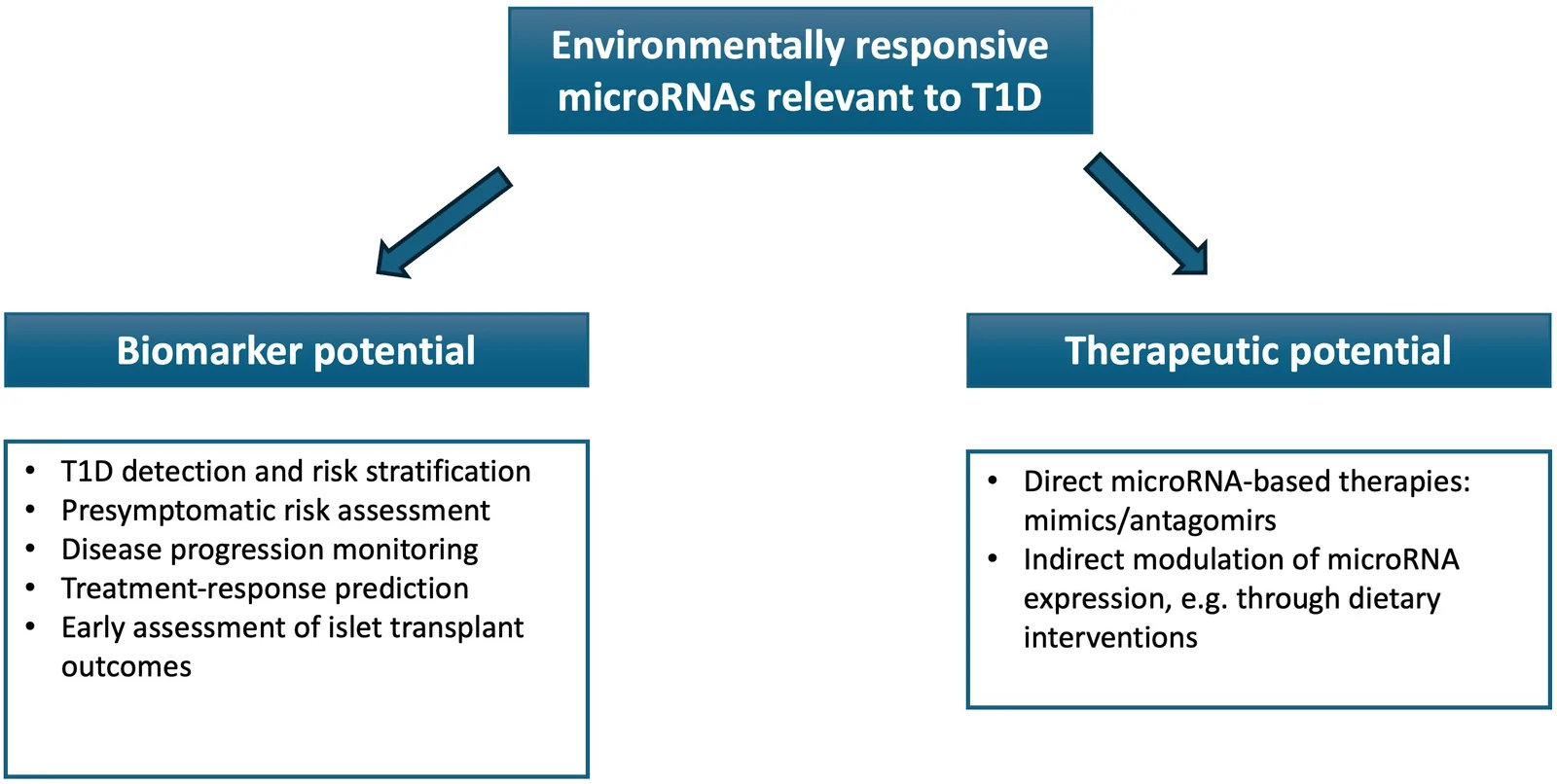
Clinical potential of environmentally responsive microRNAs in T1D. Environmentally responsive microRNAs relevant to T1D may have potential as biomarkers for early disease detection and risk stratification, presymptomatic risk assessment, monitoring of disease progression, prediction of treatment response and early assessment of islet transplant outcomes. Their therapeutic potential may involve direct microRNA-based approaches, including mimics and antagomirs, as well as indirect modulation of microRNA expression through dietary interventions. Direct microRNA-based therapies are not currently approved for T1D, and whether dietary modulation of disease-relevant microRNAs can influence T1D progression in humans remains unknown.

## 7 Discussion

Environmental exposures and microRNA dysregulation have both been implicated in T1D, but they have usually been studied separately. A strength of this review is the integration of these two areas and the comparison of evidence across different environmental exposures, tissues and experimental models. Taken together, the available studies support the possibility that environmentally responsive microRNAs contribute to the regulation of immune, inflammatory and β-cell pathways relevant to T1D. At the same time, the strength of the evidence varies considerably, and the data do not yet support a single established environment–microRNA pathway.

The most directly T1D-relevant experimental evidence comes from studies in NOD mice and human pancreatic islets or β-cells. Gut microbiota disruption in NOD mice, for example, was accompanied by changes in intestinal microRNAs and altered T1D development, while Coxsackievirus studies showed marked microRNA dysregulation in human islets and β-cell models. These studies provide evidence that environmental stimuli relevant to T1D can alter microRNA expression in biologically relevant settings. However, whether the resulting microRNA changes mediate the effect of the exposure on disease development has generally not been tested directly. Other associations, particularly those involving dietary factors, pollutants and maternal conditions, are supported mainly by non-T1D tissues or populations and therefore provide more indirect evidence.

An important finding of this review is the recurrence of several microRNAs across otherwise distinct environmental exposures. In particular, miR-155, miR-146a and miR-375 were repeatedly identified in association with different factors linked to T1D. This pattern suggests that they may represent points of convergence between environmental influences and pathways involved in immune regulation and β-cell function. This recurrence may partly reflect their biological positioning in pathways repeatedly engaged in T1D-related processes - miR-155 is closely linked to inflammatory signaling and β-cell injury (35, 65), miR-146a participates in the regulation of inflammatory signaling and β-cell responses to pro-inflammatory cytokines (77, 81) and miR-375 is closely involved in β-cell glucose metabolism, insulin secretion and survival (71, 72).

Their recurrence across different settings also provides a rationale for prioritizing these microRNAs in future experimental studies. Nevertheless, the direction of dysregulation and the tissue context are not always consistent, indicating that their effects are likely to depend on the biological setting rather than reflect a single common mechanism.

miR-146a provides a particularly clear example of this context dependence. Reduced miR-146a-5p expression has been observed following PM2.5 exposure and reduced miR-146a expression has also been reported in individuals with T1D (53, 127). Reduced miR-146a may weaken its negative-feedback control of inflammatory signaling through IRAK1 and TRAF6, potentially favoring an excessive inflammatory response (52, 53). In contrast, enteroviral infection can induce miR-146a expression: persistent CVB4 infection increased miR-146a-5p in pancreatic ductal cells (79), while experimental enterovirus infection showed that miR-146a induction suppressed IRAK1 and TRAF6, reduced type I interferon production and facilitated viral propagation (81). Pro-inflammatory cytokines also increased miR-146a expression in pancreatic β-cell models, where miR-146a overexpression promoted apoptosis and its inhibition was partially protective (77). Thus, apparently opposite changes in miR-146a expression are not necessarily contradictory, but may reflect distinct regulatory functions depending on the exposure, tissue and inflammatory or antiviral context.

The potential relevance of microRNAs also extends to their clinical application. Circulating microRNA signatures have shown promise for T1D discrimination, risk stratification and prediction of responses to therapeutic or biological interventions. Recent findings from PREDICT T1D further support the feasibility of combining multiple circulating microRNAs into clinically informative signatures across geographically diverse populations (132). Importantly, biomarker value does not require a microRNA to be a causal mediator of disease. Some circulating changes may instead reflect β-cell injury, immune activation or other disease-related processes, which may still make them clinically useful.

The overlap between environmental responsiveness, involvement in T1D-related pathways and emerging biomarker potential makes selected microRNAs particularly interesting for further study. It also raises the possibility that environmental modification could eventually influence disease-relevant microRNA pathways. At present, however, this remains a testable hypothesis rather than an established preventive or therapeutic strategy. No human study has yet shown that modifying a specific environmental exposure alters the corresponding microRNA pathway in a way that changes progression to T1D.

The next step should therefore be to test these relationships more directly. Longitudinal studies measuring defined environmental exposures together with microRNA profiles before and during the development of islet autoimmunity would help establish their temporal relationship. Sex-specific microRNA responses also warrant further investigation, as the association between maternal pre-pregnancy BMI and placental miR-146a in the ENVIRONAGE cohort was observed in female but not male offspring. Circulating signatures should be linked, where possible, to changes in pancreatic or immune-cell compartments. Finally, candidate microRNAs identified across several exposures should be tested using perturbation and rescue experiments in T1D-relevant models to determine whether they are only markers of environmental responses or functionally contribute to them. Such studies would allow the recurrent associations identified in this review to be moved from biological plausibility to functional validation and, potentially, clinical relevance.

## 8 Limitations

Several limitations should be considered when interpreting the evidence discussed in this review. The included studies differ substantially in design, study populations, biological material, experimental models, assessment of environmental exposures and methods used for microRNA quantification. This heterogeneity makes direct comparison between studies difficult and may partly explain differences in the reported findings.

Another issue is the inconsistent reporting of microRNA strand identity. Several primary studies did not specify whether the reported microRNA represented the 5p or 3p arm. In these cases, we retained the nomenclature used by the original authors and did not assign an arm. Therefore, concordance between studies cannot always be assessed at the strand level.

Major limitation is the lack of studies directly examining environmental exposure, microRNA changes and T1D within the same population. Accordingly, none of the environmental factor–microRNA associations identified in this review met the criteria for evidence category A in **Table 1**, defined as direct evidence obtained in individuals with T1D or established islet autoimmunity. In many cases, an environmental factor was linked to a specific microRNA in one study, while its possible relevance to T1D was inferred from experimental or mechanistic studies. Therefore, the available evidence supports a possible biological link, but does not demonstrate that microRNAs mediate the effects of environmental exposures on T1D development.

Much of the available evidence is also cross-sectional or derived from experimental models, which limits conclusions about the timing and direction of these associations. Prospective studies jointly assessing defined environmental exposures and microRNA profiles during progression from islet autoimmunity to clinical T1D are lacking. In addition, circulating microRNAs may originate from different tissues and cell types, making it difficult to determine whether observed changes reflect β-cell injury, immune activation, environmental exposure or a combination of these processes.

Finally, this is a narrative rather than a systematic review. The literature was therefore not selected or evaluated using a formal systematic-review framework, and the included studies reflect the predefined scope of the review.
